# A rare presentation of penile keloids after traditional circumcision: Case report

**DOI:** 10.1016/j.ijscr.2019.05.059

**Published:** 2019-06-11

**Authors:** Laura Siyabonga Madalitso Cappuyns, Devor Kumiponjera, Simbarashe Gift Mungazi

**Affiliations:** a Queen Elizabeth Central Hospital, Blantyre, Malawi; b Department of Surgery and Anaesthetics, National University of Science and Technology, Bulawayo, Zimbabwe

**Keywords:** Penile keloids, Traditional circumcision, Corticosteroid, Case report

## Abstract

**Introduction:**

Only few cases of penile keloids have been reported in the literature. There have been no published reports on penile keloids following traditional circumcision in Africa.

**Case report:**

We present a case of a 13-year-old boy with penile keloids following traditional circumcision. The patient was successfully treated with complete excision of the keloid followed by a course of adjuvant treatment with corticosteroid injection.

**Discussion:**

Penile keloids are a rare complication despite penile surgery being common. Male circumcision is a standard operation performed worldwide for medical, religious as well as cultural reasons. Traditional circumcision continues to be practised in many African countries. Keloids are a benign hyperproliferative growth of scar tissue that can complicate wound healing. Successful treatment is classically multimodal.

**Conclusion:**

This case highlights one of the more serious complications of circumcision. Although penile keloids are rare, the techniques involved in traditional circumcision potentially predispose to keloid formation in a genetically prone population.

## Highlights

• Penile keloids are rare.
• Traditional circumcision a risk factor.
• Treatment is multimodal.

## 1 Introduction

This work is reported in line with the Surgical Case Report Guidelines (SCARE) criteria [1]. Traditional male circumcision in Malawi is commonly practiced amongst the Yao ethnic group and Muslim communities in the south of the country. The Yao perform circumcision as part of an intricate ceremony called *Jando*. This socio-cultural practice is considered essential for boys at puberty as a rite of passage into adulthood [2]. The circumcision procedure is usually done by a traditionalist using unconventional techniques. Complication rates following traditional circumcision are known to be significantly higher than for medical circumcision [3]. There however have been no published cases on penile keloids following traditional circumcision in Africa.

Keloids are benign overgrowth of scar tissue that occur in genetically predisposed individuals. Keloids are commonly seen in areas such as the shoulders, sternal area, upper back, posterior neck and earlobes [4,5]. Penile keloids are remarkably rare. It is known that in individuals who are prone to keloids, surgical techniques that result in excessive skin tension, delayed wound healing, infection and foreign body reaction further increase the likelihood of keloid formation [6,7]. Multiple treatment modalities exist for keloids. No single treatment has proven widely effective and so multimodal treatment is often implored [4]. Penile keloids can be especially challenging to treat [6].

## 2 Case report

**Fig. 1:**
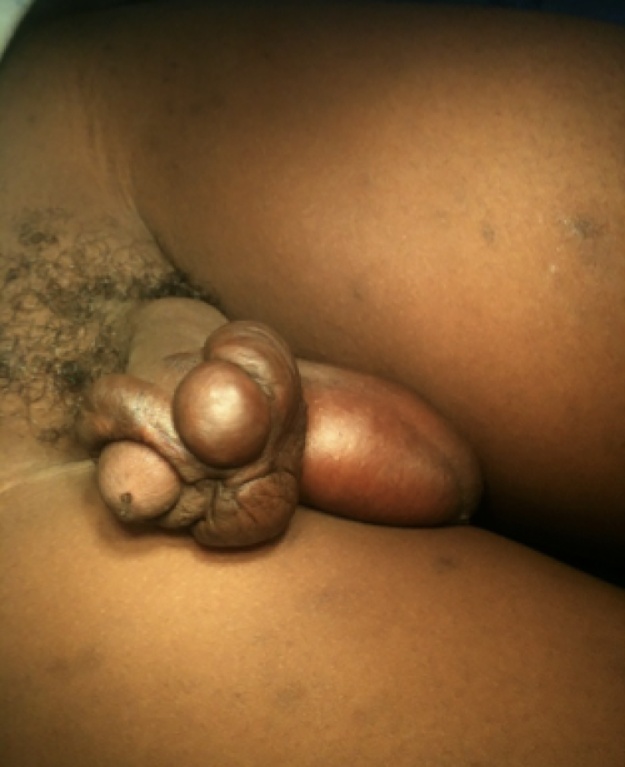
Penile keloids.

**Fig. 2:**
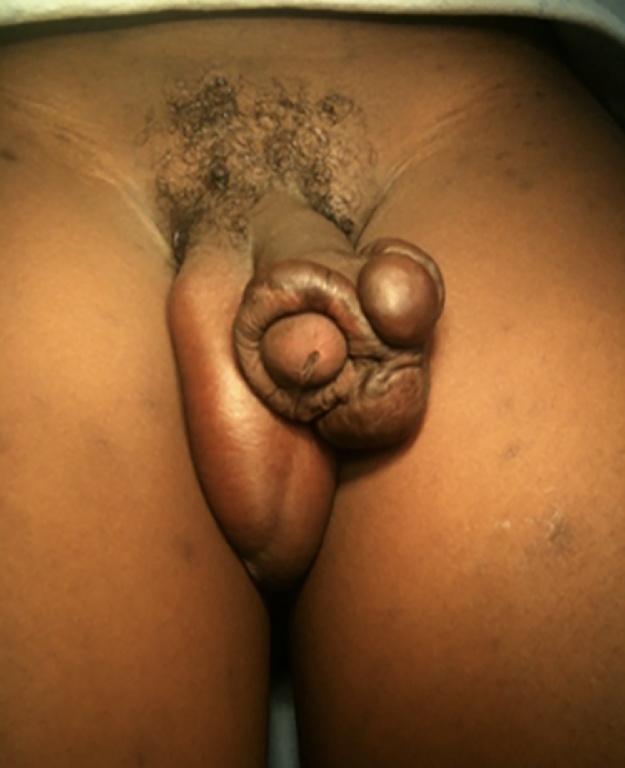
Penile keloids.

A 13-year-old boy was referred to our plastic surgery clinic with complaints of a slow-growing enlarged pruritic mass on his penis for over a year. He had had traditional male circumcision one year prior to his presentation. We asked the patient’s father to describe the process of traditional circumcision so that we could better understand the likely cause of the presentation. The procedure was done at a traditional initiation ceremony for boys which takes place around the same time every year. The process involved use of a razor blade to cut off the foreskin, without use of an anaesthetic. Haemostasis was achieved using pressure by wrapping material such as leaves and bamboo twigs around the wound. Finally a herbal paste was applied to the wound. He further went on to tell us that the recovery process had been slightly delayed compared to other boys in the same cohort. The patient’s past medical history was unremarkable. There was no family history of keloids according to the father. Examination showed a large irregularly shaped keloid along the circumference of the coronal sulcus, it measured approximately 6 cm broad and 5 cm thick in its widest dimensions (see Fig. 1, Fig. 2) The patient also had other areas of keloids over his chest, both shoulders and back sustained from ritual ‘tattooing’ around the same period of the circumcision.

Consent for surgery and photography was sought from the patient’s father as the patient was considered a minor. The patient was scheduled for elective surgery under general anaesthesia. Surgery was performed by a consultant Plastic surgeon. The surgical procedure involved complete, circumferential excision of the keloid tissue to the level of dartos fascia. The circumcision-like wound was minimally undermined to allow a tension-free closure. Haemostasis was achieved with electrocautery. Single layer closure was done with nylon 5.0 sutures. Corticosteroid (1 ml of triamcinolone acetonide 40 mg/ml) was injected into the wound edges after skin closure. Standard dressings were used which included sterile Vaseline gauze and dry gauze. Corticosteroid was also injected into the keloids in other sites while patient was under anaesthesia. The peri-operative period was uneventful. The patient was sent home for review on the third post-operative day. With the view of his propensity for keloid formation he was given comprehensive advice to avoid all forms of cutaneous trauma.

**Fig. 3:**
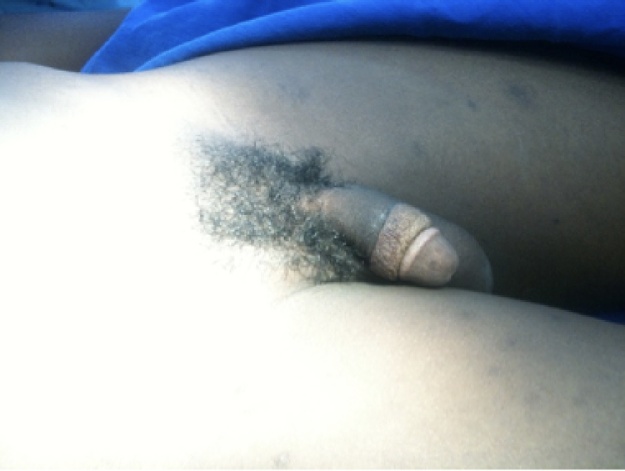
Two months post operatively.

Sutures were removed on the 14^th^ post-operative day and a second dose of corticosteroid was injected. Wound healing was uncomplicated. The patient then returned for repeat injection every 6 weeks for a period of 6 months (see Fig. 3). There was no recurrence after 1 year although the ventral aspect had a very mild degree of hypertrophic scarring. The keloids on his shoulders, chest and back also showed remarkable flattening of the scars. All injected area had minor degrees of hypo-pigmentation within the scar extending to a small surrounding circumference of normal skin. This did not bother the patient and he was assured that it was likely to improve after cessation of injections.

**Fig. 4:**
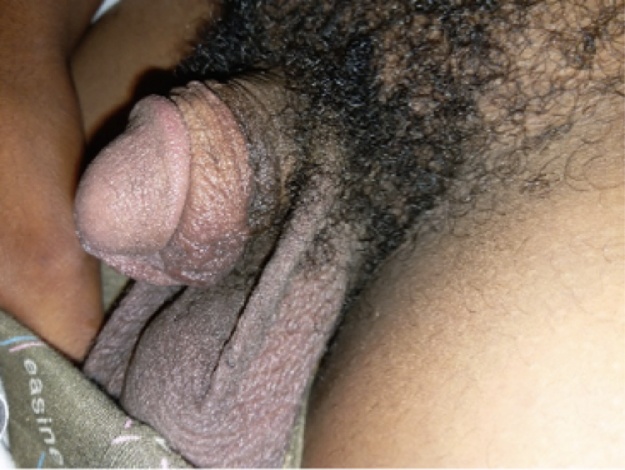
Three years post operatively.

The patient was reviewed 3 years later with complaints of pruritus along the ventral surface of the penis (see Fig. 4). There was no significant change in the size of the scar and the patient was treated with another course of 6 weekly corticosteroid injections successfully. He was discharged from clinic and for further follow up on request. He was grateful for the assistance.

## 3 Discussion

Keloids histologically consist of excessive proliferation of dermal collagen and extracellular matrix. Keloids are differentiated from hypertrophic scars in that they extend beyond the area of the original wound margin and spontaneous resolution does not occur [4,7]. Keloids can grow to large sizes and encroach on normal skin. They are commonly complicated by pruritus, tenderness, burning sensation, ulceration and secondary infection [8].

Treatment for keloids commonly includes surgical excision, intralesional corticosteroid injection, radiotherapy, cryotherapy, laser therapy, pressure therapy and silicone sheets. Surgical excision when used alone has recurrence rates of up to 100% [6,9]. Intralesional corticosteroids are first-line treatment for keloids. They decrease inflammation, increase vasoconstriction, inhibit fibroblast proliferation and relieve symptoms associated with keloids such as pruritus. Combination therapy has been shown to reduce recurrence to below 50% [6,9]. Steroid treatment carries adverse effects such as subcutaneous atrophy, telangiectasis, hypopigmentation as well as systemic side effects [4]. Different treatment protocols using corticosteroid injection exist for keloids. For keloid treatment in our centre we administer the first injection of corticosteroid triamcinolone acetonamide into the incision at end of surgery, then after 2 weeks and then 6 weekly thereafter, for a period of 6–7 months. To our knowledge, there are no published reports on which corticosteroid dose or injection protocol is superior.

Traditional male circumcision in Malawi performed by the Yao ethnic group is a practice that has existed for decades. From what was described in the case report, we can deduce that traditional circumcision is likely to be at risk of various complications. The technique is non-sterile and use of the herbal paste is likely to contribute to further contamination of the wound. It is likely that use of such unconventional methods would result in more infection, delayed wound healing and foreign body reaction, all of which increase the risk of keloid formation [6]. This was the likely scenario with our patient.

In 2007, the World Health Organisation (WHO) and the United Nations Program on HIV/AIDS (UNAID) made recommendations that male circumcision be recognized as an additional important intervention to reduce the risk of heterosexually acquired HIV infection in men [10]. These recommendations were based on strong evidence from three randomized controlled trials which took place in three different countries in Africa. The studies showed that male circumcision reduces the risk of heterosexually acquired HIV infection in men by approximately 60%. In addition to being protective against HIV infection, there is significant evidence that suggests that circumcision protects males from penile carcinoma, urinary tract infections, and ulcerative sexually transmitted diseases [11]. WHO/ UNAIDS have placed emphasis on the need for quality and safe circumcision services, identifying that serious complications can arise if circumcision is undertaken in unhygienic settings by poorly trained providers or with inadequate instruments. In the year 2007, it was estimated that around 30% of the world’s male population were circumcised [10]. The Joint United Nations Program on HIV/AIDS (UNAIDS) have implemented free voluntary medical male circumcision (VMMC) in Malawi and other Sub-Saharan countries since 2008 as one strategy for reducing the spread of HIV/AIDS [3]. Despite this free service, ritual circumcision continues to be a common practice and is considered an important socio-cultural entity by those that practice it [3]. Unfortunately, with poor health seeking behaviour amongst the rural population in Malawi, it is not known if other cases of penile keloids exist in the community.

Penile keloids can cause sexual dysfunction, somatic discomfort, and mental anxiety and therefore complete removal is the goal of treatment [6]. According to the reports published on penile keloids, surgical excision and intralesional steroid injection are the preferred treatments [6]. Mechanical pressure and silicone sheets are not feasible to apply on the penis [6]. Radiotherapy is contraindicated due to the proximity of the penis to the gonads [7]. Our patient was successfully treated with complete excision and a course of corticosteroid injection. To minimise foreign body reaction, we opted to use non-absorbable nylon suture which was removed when wound healing was adequate.

## 4 Conclusion

We present a case of a rare complication of keloid formation following traditional male circumcision and successful treatment with surgery and steroid injection. We also highlight one potential complications of traditional circumcision. In a community with poor health seeking behaviour, it is difficult to ascertain the true incidence of this rare predicament.

## Conflicts of interest

There is no conflict of interest.

## Sources of funding

There is no funding for the case report.

## Ethical approval

Ethical approval was exempted by the institution.

## Consent

Signed consent obtained from the patient’s guardian.

## Author contribution

Laura Siyabonga Madalitso Cappuyns- case report design, subject research, consent and writing.

Devor Kumiponjera- case report design, subject research, writing.

Simbarashe Gift Mungazi - case report design, subject research and writing.

## Registration of research studies

Not applicable. This is a case report with no recruitment of patients.

## Guarantor

L.S.M. Cappuyns.

## Provenance and peer review

Not commissioned, externally peer-reviewed.
